# Genome-wide association reveals host-specific genomic traits in *Escherichia coli*

**DOI:** 10.1186/s12915-023-01562-w

**Published:** 2023-04-11

**Authors:** Sumeet K. Tiwari, Boas C. L. van der Putten, Thilo M. Fuchs, Trung N. Vinh, Martin Bootsma, Rik Oldenkamp, Roberto La Ragione, Sebastien Matamoros, Ngo T. Hoa, Christian Berens, Joy Leng, Julio Álvarez, Marta Ferrandis-Vila, Jenny M. Ritchie, Angelika Fruth, Stefan Schwarz, Lucas Domínguez, María Ugarte-Ruiz, Astrid Bethe, Charlotte Huber, Vanessa Johanns, Ivonne Stamm, Lothar H. Wieler, Christa Ewers, Amanda Fivian-Hughes, Herbert Schmidt, Christian Menge, Torsten Semmler, Constance Schultsz

**Affiliations:** 1 Robert Koch Institute, Genome Sequencing and Genomic Epidemiology, Berlin, Germany; 2 Quadram Institute Bioscience, Gut Microbes and Health Institute Strategic Program, Norwich Research Park, Norwich, UK; 3 Department of Global Health, Amsterdam Institute for Global Health and Development, Amsterdam UMC, University of Amsterdam, Amsterdam, Netherlands; 4 Department of Medical Microbiology, Amsterdam UMC, University of Amsterdam, Amsterdam, Netherlands; 5 Friedrich-Loeffler-Institut, Institute of Molecular Pathogenesis, Jena, Germany; 6 Oxford University Clinical Research Unit, Ho Chi Minh City, Vietnam; 7 Faculty of Veterinary Medicine, College of Agriculture, Can Tho University, Can Tho, Vietnam; 8 UMC Utrecht, Utrecht, Netherlands; 9 Amsterdam Institute for Life and Environment, Faculty of Science, Vrije Universiteit Amsterdam, Amsterdam, Netherlands; 10 Department of Comparative Biomedical Sciences, School of Veterinary Medicine, University of Surrey, Guildford, UK; 11 Department of Microbial Sciences, School of Biosciences, University of Surrey, Guildford, UK; 12 Tropical medicine and global health, Nuffield Department of Medicine, University of Oxford, Oxford, OX3 7BN UK; 13 Microbiology- Parasitology Unit, Biomedical Research Center and Microbiology Department, Pham Ngoc Thach University of Medicine, Ho Chi Minh City, Vietnam; 14 VISAVET Health Surveillance Centre, Complutense University of Madrid, Madrid, Spain; 15 Department of Animal Health, Faculty of Veterinary Medicine, Complutense University of Madrid, Madrid, Spain; 16 Robert Koch Institute, Enteropathogenic Bacteria and Legionella, Wernigerode, Germany; 17 Institute of Microbiology and Epizootics, Freie Universität Berlin, Berlin, Germany; 18 Veterinary Centre for Resistance Research (TZR), Freie Universität Berlin, Berlin, Germany; 19 Robert Koch Institute, Advanced Light and Electron Microscopy, Berlin, Germany; 20 Vet Med Labor GmbH, Division of IDEXX Laboratories, Kornwestheim, Germany; 21 Robert Koch Institute, Berlin, Germany; 22 Institute of Hygiene and Infectious Diseases of Animals, Giessen, Germany; 23 Institute of Food Science and Biotechnology, Department of Food Microbiology and Hygiene, University of Hohenheim, Stuttgart, Germany

**Keywords:** *Escherichia coli*, GWAS, Host-specificity, Sialic acid

## Abstract

**Background:**

*Escherichia coli* is an opportunistic pathogen which colonizes various host species. However, to what extent genetic lineages of *E. coli* are adapted or restricted to specific hosts and the genomic determinants of such adaptation or restriction is poorly understood.

**Results:**

We randomly sampled *E. coli* isolates from four countries (Germany, UK, Spain, and Vietnam), obtained from five host species (human, pig, cattle, chicken, and wild boar) over 16 years, from both healthy and diseased hosts, to construct a collection of 1198 whole-genome sequenced *E. coli* isolates. We identified associations between specific *E. coli* lineages and the host from which they were isolated. A genome-wide association study (GWAS) identified several *E. coli* genes that were associated with human, cattle, or chicken hosts, whereas no genes associated with the pig host could be found. In silico characterization of nine contiguous genes (collectively designated as *nan-9*) associated with the human host indicated that these genes are involved in the metabolism of sialic acids (Sia). In contrast, the previously described sialic acid regulon known as sialoregulon (i.e. *nanRATEK-yhcH*, *nanXY*, and *nanCMS*) was not associated with any host species. In vitro growth experiments with a Δ*nan-9 E. coli* mutant strain, using the sialic acids 5-*N*-acetylneuraminic acid (Neu5Ac) and *N*-glycolylneuraminic acid (Neu5Gc) as sole carbon source, showed impaired growth behaviour compared to the wild-type.

**Conclusions:**

This study provides an extensive analysis of genetic determinants which may contribute to host specificity in *E. coli*. Our findings should inform risk analysis and epidemiological monitoring of (antimicrobial resistant) *E. coli*.

**Supplementary Information:**

The online version contains supplementary material available at 10.1186/s12915-023-01562-w.

## Background

*Escherichia coli* is a Gram-negative bacterium that can colonize various hosts, including humans, cattle, chickens, and pigs [1]. Because of its host promiscuity, the *E. coli* species as a whole can act as a reservoir for genes encoding antimicrobial resistance (AMR) [2] that can be transmitted between different animal host species. The likelihood that *E. coli* and its AMR encoding genes persist in a new host after transmission depends on multiple factors [3, 4]. For example, small changes in metabolic pathways may enable particular *E. coli* clones within the species to colonize a certain host more efficiently [1]. Several studies have suggested that highly successful *E. coli* clones, such as those of sequence type 131 (ST131) [5, 6] or clones belonging to clonal complex 87 (ST58 and ST155), facilitate the spread of AMR in the human population [7], whilst other studies have shown that different lineages of AMR *E. coli* vary in their ability to spread [8]. These latter findings indicate that AMR genes, at least to some extent, can hitchhike on bacterial strains that are specifically equipped to colonize a given host. Beyond classical virulence or adhesion factors, genetic and functional traits defining different degrees of host adaptation [3, 9], thereby indirectly impacting on the spread of AMR between host species, have not been identified thus far.

Comparative genomics approaches have previously revealed the signatures of host-adaptation in bacterial genomes [10–13]. The emergence of large-scale bacterial genome-wide association studies (GWAS) allowed the detection of genes or genomic variants that are associated with antimicrobial resistance, pathogenicity, and host-adaptive traits [14–16]. Here, we have applied population-based bacterial GWAS to identify host-associated genomic determinants in a diverse panel of 1198 *E. coli* isolates, irrespective of their AMR profile. Isolates were recovered from five different host species, including healthy and diseased individuals from four different countries in two continents over 16 years. We analysed host colonization patterns associated with *E. coli* lineages and applied gene-based and *k-mer*-based bacterial GWAS to identify host-associated genomic determinants in the *E. coli* accessory genome.

## Results

### Data collection

After WGS quality control, sequences of 14 isolates were excluded because of poor quality. One additional isolate was excluded since this isolate was identified as *Escherichia marmotae* (formerly cryptic clade V) [17, 18], a species commonly mistaken for *E. coli*. Our final collection comprised 1198 *E. coli* whole-genome sequences with associated metadata (Additional file 2: Table S1), including 8 cryptic clade I isolates, which were included as *E. coli* based on the recommended species cut-off of 95–96% average nucleotide identity [17]. Our collection consisted of 22.1% (*n* = 265) cattle, 28.1% (*n* = 337) chicken, 27.3% (*n* = 327) human, 20.3% (*n* = 240) pigs, and 2.4% (*n* = 29) wild boar isolates (Additional file 1: Fig. S1A). Fifty-one percent (*n* = 612), 19.4% (*n* = 233), 14.5% (*n* = 174), and 14.9% (*n* = 179) of these isolates were from Germany, Spain, the UK, and Vietnam, respectively (Additional file 1: Fig. S1A). Chicken isolates were from all four countries, human isolates from Germany, the UK, and Vietnam; pig isolates from Germany, Spain, and Vietnam; and cattle isolates from Germany and Spain. Only Spain provided wild boar isolates. In total, 35.5% (*n* = 426) of the isolates were from hosts with reported disease, whereas 62.0% (*n* = 743) were from hosts without reported disease. Host health status was unknown for the wild boar isolates (2.4%, *n* = 29). Of the 1198 isolates analysed, 1140 were grouped into 358 different STs, and 58 could not be assigned to any known ST. The population structure of the collection closely resembles that of the ECOR collection [19], indicating that it represents most of the known diversity of *E. coli sensu stricto* (Additional file 1: Fig. S2).

### Pan-genome analysis

**Fig. 1:**
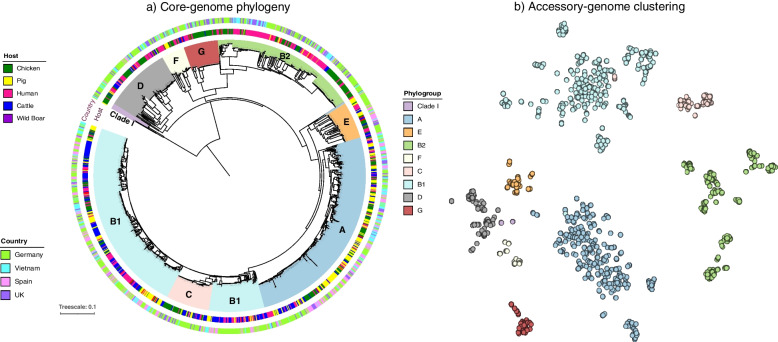
Distribution of 1198 *E. coli* isolates with host species by **a** core-genome phylogeny and **b** clustering based on accessory gene content. Clades on the phylogeny represent phylogroups, the inner-ring represents host-species, and the outer ring indicates the geographical region. Each bubble in accessory gene clustering represents individual *E. coli* strains coloured according to phylogroups

The pan-genome of the 1198 *E. coli* isolates consisted of 77,130 genes, of which 1956 genes belonged to the core genome (i.e. present in at least 99% of the isolates). Bayesian analysis of population structure (BAPS) revealed 14 BAPS clusters using core-genome alignment (Additional file 1: Fig. S3). Furthermore, most of the isolates were assigned to phylogroups B1 (*n* = 366, 30.55%), A (*n* = 313, 26.12%), and B2 (*n* = 213, 17.77%). The remaining isolates were distributed among phylogroups D (*n* = 97, 8.09%), E (*n* = 55, 4.59%), G (*n* = 49, 4.09%), F (*n* = 35, 2.92%), C (*n* = 60, 5.0%), and clade I (*n* = 8, 0.6%). The distribution of phylogroups, country, and host on a maximum likelihood (ML) tree based on 110,920 core-genome SNPs is shown in Fig. 1a. Clustering of isolates based on core gene alignment and accessory gene profile appeared to correlate with phylogroup (Fig. 1a, b) and BAPS cluster, as expected (Additional file 1: Fig S3). The *χ*^2^-tests for independence revealed a positive correlation between host species and phylogroups (*p* < 2.2e^−16^, df = 32). This indicates the enrichment of certain hosts among phylogroups, such as B1 (with cattle), A (with pigs), B2 (with humans and chickens), and G (with chickens) within our collection (Additional file 1: Fig. S1B).

A minimum spanning tree was built on the allelic profiles of 358 (*n* = 1140 isolates) known STs and 58 isolates belonging to unknown STs using GrapeTree [20] along with the host distribution (Additional file 1: Fig. S4). Several sequence types, of which at least ten isolates were available, appeared to be linked with certain host species. ST33 (*n* = 10/10, 10 human isolates out of all 10 isolates), ST73 (*n* = 11/17), ST131 (*n* = 37/42) and ST1193 (*n* = 12/12) were associated with a human host. ST131 was also found in chickens (*n* = 4/42) and a pig (*n* = 1/42) in this collection. ST23 (*n* = 18/22), ST95 (*n* = 25/31), ST115 (*n* = 11/11), ST117 (*n* = 30/33), ST140 (*n* = 19/20), and ST752 (*n* = 29/30) were associated with the chicken host.

### GWAS

The genome-wide association analysis was performed on 1169 *E. coli* isolates from cattle, chickens, humans, and pigs using *k-mers* as well as a pan-genome matrix of gene presence. The 29 wild boar isolates were excluded because of their small group size. The thresholds used to filter *k-mers* or genes can be found in Additional file 1.

GWAS detected the association of 11,869, 3050, and 111,895 *k-mers* with a human, cattle, or chicken host, respectively. These *k-mers* accounted for 262, 69, and 1167 genes, respectively (Additional file 2: Table S2). The host-associated genes identified through the *k-mer* approach were further mapped back to their corresponding orthologous group in the pan-genome.

GWAS using the pan-genome gene presence matrix revealed a positive association of 28, 12, and 145 genes with a human, cattle, or chicken host.

**Fig. 2:**
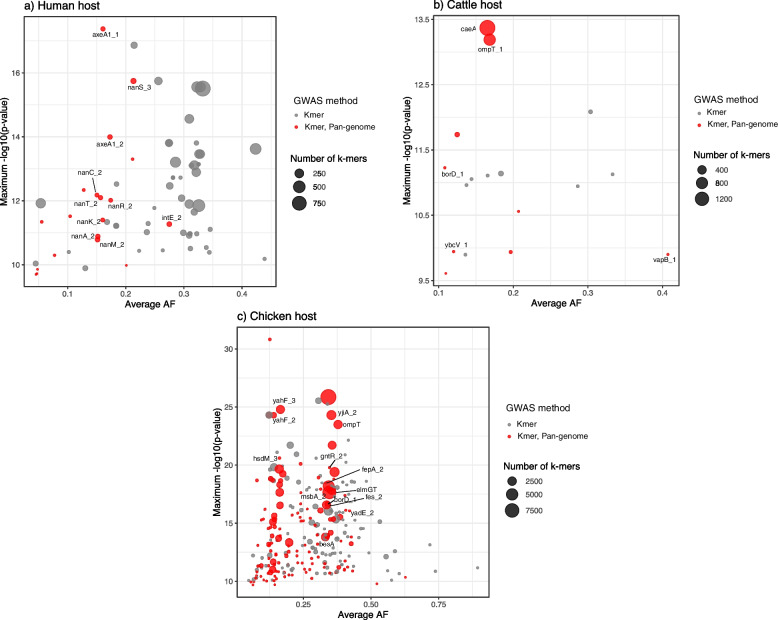
Plots representing the *E. coli* genes or gene variants associated with the **a** Human host, **b** Cattle host, and **c** Chicken host. The bubble size represents the number of *k-mers* mapped to a specific gene. The bubbles in red indicate genes which came classified as significant by both GWAS approaches (using *k-mers* and pan-genome) and the bubbles in grey indicate significance by GWAS using *k-mers* only

There were 19, 9, and 137 accessory genes of *E. coli* which were reported to be associated with a human, cattle, or chicken host, respectively, by both GWAS methods (Fig. 2, Additional file 2: Table S2). These genes were subjected to/chosen for further downstream analysis. Neither GWAS approach identified any genes as being associated with the pig host.

### Association of novel *nan* genes with human host

**Fig. 3:**
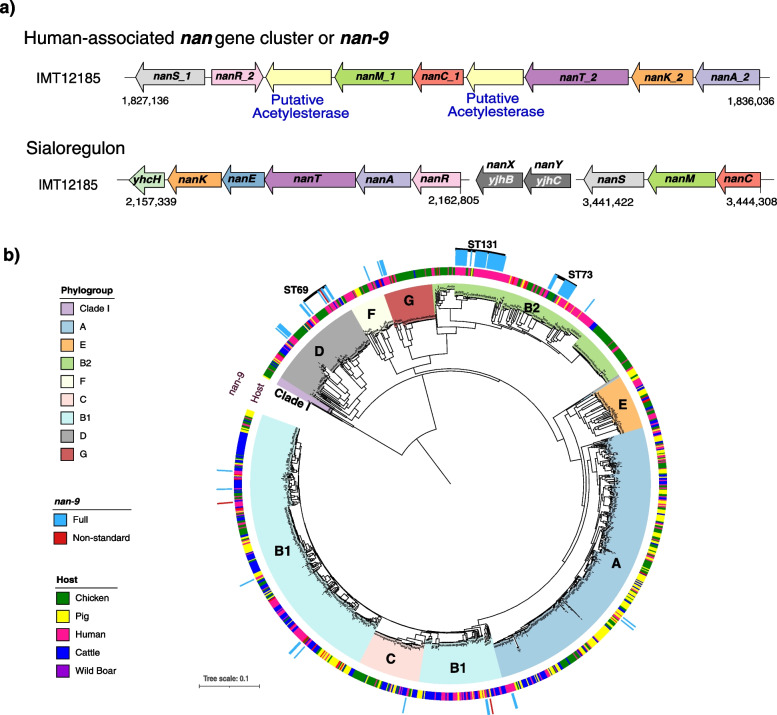
**a** Genetic architecture of the human-associated *nan* gene cluster (*nan-9*) and the sialoregulon on the complete genome of the strain IMT12185. The strain lacks the *nanXY* genes of the sialoregulon. **b** Distribution of the *nan-9* cluster on core-genome phylogeny marked with STs with higher prevalence

GWAS revealed a strong association of nine contiguous genes, assigned to the group of *nan* genes, with the human host (Fig. 2a). Seven of these genes were annotated in silico as *nan* genes (Fig. 3a), and the remaining two genes were annotated as being similar to *axeA1* of *Prevotella ruminicola* ATCC 19189 (Uniprot accession D5EV35). However, translated amino acid sequences of these *axeA1*-like genes only shared 19–20% similarity with the AxeA1 reference sequence. Further investigation with EggNOG and CD-search revealed an acetylesterase/lipase-encoding region (COG0657) in both genes and confirmed the *nan* gene annotations. Previous evidence and the genomic location (i.e. between the *nan* genes; Fig. 3a) suggest that these genes encode potential acetyl-esterases and may be analogous to sialyl esterases (NanS) [21]. Hence, these nine novel *nan* genes are collectively termed “human-associated *nan* gene cluster (*nan*-9)” (Fig. 3a).

Distinct *nan* genes are present in *E. coli* and are also known as the sialoregulon (*nanRATEK-yhcH*, *nanXY [yjhBC]*, and *nanCMS*; Fig. 3a) [22]. The sialoregulon is involved in metabolism of sialic acids [23–25], a diverse group of nine-carbon sugars, abundant in the glycocalyx of many animal tissues [26, 27]. Sialic acids present on mucin proteins in the human gut are an essential energy source for many intestinal bacteria [28]. The proteins encoded by the seven genes of *nan-*9 (i.e. *nanAKTCMRS*) share 45–64% similarity with the corresponding *nan* genes of the sialoregulon in *E. coli* or the recently described phage-encoded *nanS*-p genes of enterohemorrhagic *E. coli* [29]. Both the human-associated *nan* gene cluster and the sialoregulon are located on the bacterial chromosome. The human-associated *nan* gene cluster was found in 7% of our isolate collection, whereas the genes comprising the sialoregulon, i.e. *nanXY* was identified in ~15% of isolates, *nanCMS* in ~93% of isolates, whilst *nanRATEK-yhcH* was found in almost all (> 99%) isolates.

The *nan-*9 cluster was detected in 86 isolates, mainly from phylogroups B2 and D (Fig. 3b) and predominantly in isolates belonging to ST131, ST73, and ST69, both in our collection as well as across 17,994 *E. coli* genomes present in the RefSeq database (Table 1). The order and orientation of genes in the human-associated *nan* gene cluster were found to be identical in 82 out of 86 isolates (Additional file 1: Fig. S5). In 63 isolates, insertion sequence (IS) *682* was found upstream, and in 23 isolates, IS*2* was found downstream of this novel gene cluster (Additional file 1: Fig. S5).

**Table 1:** Prevalence of *nan-9* gene cluster among different STs in our collection and RefSeq collection of *E. coli* isolates

**Our collection**			**RefSeq**	
**STs**	**# Genomes**	**%**	**# Genomes**	**%**
131	35/42	83	1073/1256	85
73	14/17	82	253/372	68
69	6/31	19	155/302	51
38	3/6	50	57/196	29
10	4/109	4	66/1183	6
Other	24/993	3	512/14685	3
Total	86/1198	7	2116/17994	12

To further explore the function of the human-associated *nan-9* gene cluster, the entire cluster was knocked-out from strain IMT12185 (ST131), yielding strain IMT12185Δ*nan-9*. Correct gene deletion was confirmed through WGS. No significant differences in carbon utilization and chemical sensitivity were observed between wild-type strain IMT12185 and its mutant IMT12185Δ*nan-9* in Biolog phenotyping array experiments (PM1 and Gen III MicroPlates).

**Fig. 4:**
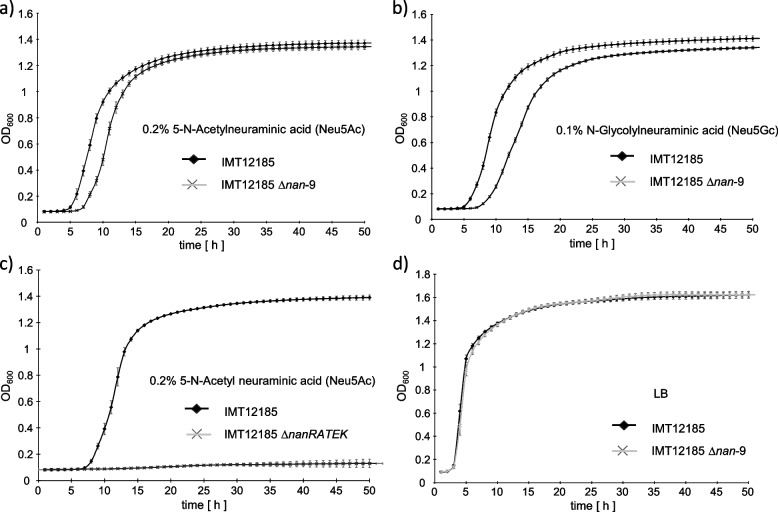
Growth curves of *E. coli* IMT12185 and its mutants derivatives in various media. **a** Growth of IMT12185 and IMT12185∆*nan*-9 in M9 minimal medium with 0.2% 5-N-acetylneuraminic acid (Neu5Ac). **b** Growth of IM12185 and IMT12185∆*nan*-9 in M9 minimal medium with 0.1% 5-N-Glycolylneuraminic acid (Neu5Gc). **c** Growth of IMT12185 and IMT12185∆*nanRATEK* in M9 minimal medium with 0.2% 5-N-acetylneuraminic acid (Neu5Ac), **d** Growth of IMT12185 and IMT12185∆*nan-9* in lysogeny broth (LB)

Deletion mutant IMT12185Δ*nan-9* was grown in minimal medium (MM) with 0.2% 5-N-acetylneuraminic acid (Neu5Ac) or with 0.1% N-glycolylneuraminic acid (Neu5Gc) as sole carbon and energy source. Neu5Ac is the most common sialic acid of the glycocalyx of both humans and other mammals, whereas Neu5Gc is absent in humans. In the presence of Neu5Ac, mutant IMT12185Δ*nan-9* grew to a maximal optical density at 600nm (OD_600_) of 1.34 comparable to that of the parental strain IMT12185 (OD_600_ = 1.37). However, mutant IMT12185Δ*nan-9* exhibited a delayed growth start of approximately three hours (Fig. 4a). When Neu5Gc was offered as substrate, mutant IMT12185Δ*nan-9* not only showed a similar growth start retardation, but also a slower growth rate and a lower maximal OD_600_ (1.31) in comparison with strain IMT12185 (OD_600_ = 1.43) (Fig. 4b). Both Neu5Ac and Neu5Gc are degraded by the enzymatic activities of the enzymes NanRATEK, of which four, namely NanRATK, are encoded by redundant genes which are located on both the loci *nanRATEK* and *nan-9*. For comparison, a mutant, which lacked the *nanRATEK* locus from the sialoregulon (IMT12185Δ*nanRATEK*), was also constructed from wild-type IMT12185. Deletion mutant IMT12185Δ*nanRATEK* was unable to grow with Neu5Ac as sole carbon and energy source (Fig. 4c), demonstrating that *nan-9* alone is not sufficient for sialic acid degradation, probably due to a lack of *nanE* in the *nan-9* gene cluster. To exclude a pleiotropic effect of the *nan-9* deletion, parental strain IMT12185 and its mutant IMT12185Δ*nan-9* were grown in LB medium. No significant difference was observed between the two growth curves (Fig. 4d). These data demonstrate that the *nan-9* determinant of strain IMT12185 is biologically functional and contributes to the degradation of the sialic acids Neu5Ac and Neu5Gc.

### Association of distinct omptins with the cattle and chicken hosts

**Fig. 5:**
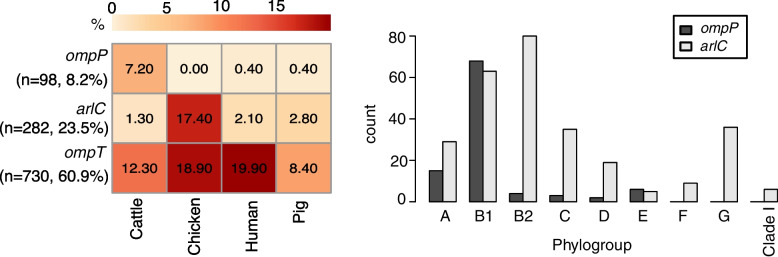
Distribution of the *ompP*, *arlC*, and *ompT* genes. Heatmap showing the prevalence (%) of omptins in different hosts, whereas the barplot indicates the count of isolates having *ompP* and *arlC* in different phylogroups

We detected multiple homologues of the *ompT* (encoding outer-membrane protease VII) gene, a member of the omptin family of proteases, in our dataset (Fig. 2b, c). Two homologues, *ompP* (UniProt accession P34210, sharing 70% amino acid identity with OmpT) and *arlC* (also referred to as *ompTp*, UniProt accession Q3L7I1, sharing 74% amino acid identity with OmpT), were found to be associated with the cattle and the chicken host, respectively (Fig. 5). In our collection, *ompP* was predominant in phylogroup B1 (*n* = 68), whereas *arlC* was found in distinct phylogroups, including B2, B1, and G (Fig. 5), and in isolates belonging to ST95 and ST117 (Additional file 2: Table S3). A similar association of *arlC* with these STs was observed in 17,994 *E. coli* genomes in the RefSeq database (Additional file 2: Table S3). Previous studies have reported an increased prevalence of *arlC* (erroneously reported there as *ompT*) in a cluster of uropathogenic *E. coli* (UPEC) and avian pathogenic *E. coli* (APEC) classified as ST95 [30]. Notably, *arlC* is associated with increased degradation of antimicrobial peptides (AMPs) in UPEC isolates [31]. OmpP is also able to degrade AMPs but displays an AMP cleavage specificity different from that of OmpT [32].

### Association of genes involved in metal acquisition with the chicken host

GWAS analysis revealed an association of the *iroBCDEN* gene cluster with the chicken host, but not with other host species included in this study (Additional file 2: Table S2). The prevalence of the *iro* gene cluster was 24.3% (*n* = 291/1,198) in our collection, of which 61.5% (*n* = 179/291) were from the chicken host. The gene cluster was found in different STs and with higher prevalence in STs such as ST117, ST95, ST23, and ST140 (Additional file 2: Table S4). The chromosomal *iroBCDEN* gene cluster was first described in *Salmonella enterica* and is involved in uptake of catecholate-type siderophores, high-affinity iron-chelating molecules contributing to bacterial survival during infection by sequestering iron [33]. In *E. coli*, this gene cluster has mainly been described in uropathogenic (UPEC) and avian pathogenic *E. coli* (APEC) and is regarded as a virulence factor [34]*.* The cluster has been reported on a chromosomal pathogenicity island, although in ExPEC, the cluster can also be located on ColV or ColBM virulence plasmids [35, 36].

## Discussion

Different clones comprising the bacterial species *Escherichia coli* can colonize various ecological niches in a diverse range of host species, with consequences ranging from a commensal lifestyle to causing intra- or extra-intestinal infectious diseases. The presence of certain adhesin and other virulence-associated genes is well known to correlate with the relative ability of *E. coli* clones to colonize the intestinal tract of certain hosts (e.g. *ecp* for humans [37], F9 fimbriae, and H7 flagellae for cattle [38, 39] or Stg fimbriae for chickens [40]). Variations in host adaptation levels and their molecular basis in *E. coli* clones presumptively realizing a commensal-like lifestyle in the reservoir host are rarely described and poorly understood as of yet [41]. Commensal *E. coli* strains may act like a carrier of AMR and a source of mobile genetic elements conferring AMR to other bacteria including pathogenic strains in a shared microbiome, e.g. in the intestinal tract of animals including humans. We therefore collated an extensive and diverse dataset to identify genetic determinants of *E. coli* host adaptation. We observed significant enrichment of specific hosts within some phylogroups and STs in our collection. Furthermore, we unveiled correlations between the likelihood of genetically related isolates having been isolated from a certain host with the possession of distinctive genetic traits. Some of these traits, e.g. the *iroBCDEN* gene cluster, have been linked to *E. coli* and *Salmonella* virulence before, whilst others, in particular the human-associated *nan* gene cluster, are novel traits and have not been implicated in the colonization and infection process of *E. coli*. Of note, the latter gene cluster encodes metabolic properties which have received little attention in bacterial infectious disease research thus far. Specific metabolic properties have been linked to the relative ability of Shiga toxin-encoding *E. coli* (STEC) to asymptomatically colonize cattle, their reservoir host [42]. Unravelling the nutrient and energy flows in the complex interplay of intestinal bacteria, the surrounding microbiome and the host may open novel avenues to control the persistence and transmission of pathogenic and/or antimicrobial resistant bacteria [43].

We employed a *k-mer* as well as gene-based bacterial GWAS, applied in previous studies to associate multiple types of genetic variation with phenotypes [44, 45]. Our approach focused on accessory genes associated with particular host species, detected through either whole gene or *k-mer* associations. Although in theory *k-mer*-based bacterial GWAS would be able to also associate SNP variation to phenotypes, this is difficult in diverse datasets such as these. If multiple sites within a gene vary in sequence, the number of possible *k-mers* from that sequence inflates quickly. If we tried to link mutations in this region to a phenotype, this would be very complicated based on *k-mers* alone which is why we focused on variation in the accessory genome. By doing so, we were able to confirm previously published host associations, indicating the validity of our approach. For example, carriage of the salmochelin operon encoded by *iroBCDEN* was previously identified as associated with increased ability of *E. coli* strains to colonize chickens [34, 46].

In addition to *iroBCDEN*, we found an association of omptin proteins (OmpP and ArlC) with chickens and cattle as hosts, respectively. Earlier studies using UPEC strains demonstrated that these proteins are associated with cleavage and inactivation of cationic antimicrobial peptides (AMPs) [31]. Because AMPs are secreted as part of the host’s innate immune response [47–49], these proteins may play a vital role in colonization. Because AMPs are also considered as alternatives to antimicrobial agents in animal farming [50–52], further investigation into the contribution of these Omp variants to host colonization as well as to resistance to exogenous AMPs is warranted.

We did not identify any significant association of *E. coli* genes with strains isolated from the pig host. Bacterial colonization of the porcine intestine by edema-disease *E. coli* (EDEC) is mediated by the ability of these bacteria to adhere to villous epithelial cells via their cytoadhesive F18 fimbriae [53]. The expression of receptors for these fimbriae on the apical enterocyte surface is inherited as a dominant trait among pigs and determines susceptibility to diseases caused by F18-fimbriated pathogenic *E. coli* [54]. Enterotoxigenic *E. coli* (ETEC) express F4 or F5 fimbriae with similar consequences [55]. However, we found only three, four, and six isolates harbouring genes for F4, F5, and F18 fimbriae, respectively. Thus, we might not have had all *E. coli* pathovars associated with pig host sufficiently present in our collection, although we did observe an association between phylogroup A and pig colonization. On the other hand, we did found many genes associated with chicken colonization. This might be due to several reasons. First, *E. coli* lineages that are typically isolated from chicken are abundant in our dataset, such as ST95 [56] and ST117 [57]. The salmochelin operon, associated with chicken in our analysis, is also prevalent in these lineages. Second, the comparison of chicken (avian species) versus human, cattle, and pig (mammalian species) may provide the best resolution of all GWAS comparisons. Due to the large evolutionary difference between mammalian and avian host species, which might result in diverse host adaptive mechanisms in *E. coli*, it is likely that certain traits of the gastrointestinal tract or other niches are shared among mammalian species but are different in avian species.

We identified a novel human host-associated *nan* gene cluster, distinct from the previously reported sialic acid (Sia) metabolic operon (*nanRATEK-yhcH*, *nanXY*, and *nanCMS) *[22]. This novel cluster was found to be conserved and abundant in ExPEC lineages, such as ST131, ST73, and ST69. The gene cluster is flanked by insertion sequences which might play a role in the horizontal exchange between different *E. coli* lineages. Knock-out in vitro studies indicated that this novel *nan-9* gene cluster contributes to catabolism of the sialic acids Neu5Ac and Neu5Gc, although it cannot replace the function of the *nanRATEK* locus which is encoded in the core genome of *E. coli*. Hence, we hypothesize that *E. coli* harbouring the *nan-9* gene cluster have an evolutionary advantage through either more efficient access to sialic acids or through access to more diverse sialic acids. The genes annotated as acetylxylan esterases are expected to represent novel sialyl esterases, as known sialyl esterases (*nanS* variants) have previously been mistaken for acetylxylan esterases [21]. Additional sialyl esterases—possibly with alternative deacetylation specificity—might provide a more efficient catabolism of acetylated sialic acids. Future studies should investigate the role of the human-associated *nan-9* gene cluster in the catabolism of differentially acetylated sialic acids and their relevance for the human host.

Approximately one third of the isolates in our dataset were obtained from diseased hosts, whilst the remaining isolates were from healthy hosts. Many of the isolates in our dataset that originate from healthy hosts belong to ExPEC lineages which are typically considered to be pathogenic. In fact, the locus most strongly associated with the human host, the *nan-9* gene cluster, is abundant in ExPEC lineages. This does not necessarily mean that the *nan-9* gene cluster is associated with pathogenicity. In fact, this observation may support the notion that these pathogenic *E. coli* are highly efficient colonizers of the human intestine [41]. Based on our results, we hypothesize that the human-associated *nan-9* gene cluster is one of the factors driving the adaptation of ExPEC to the human intestine.

## Conclusions

Our study identified several distinct genetic determinants that may promote *E. coli* adaptation to different host species and provide an adaptive advantage. These findings are important as they aid to better understand the potential outcome of transmission events of *E. coli* between host species. This is particularly relevant for controlling the spread of antimicrobial resistant commensal and zoonotic *E. coli* strains within and across human and animal populations. The data generated here can also pave the way for the improvement of risk analysis and diagnostic and monitoring efforts. More importantly, our study identified biological processes, including sialic acid catabolism, that should be investigated in more detail to better understand *E. coli* host adaptation.

## Methods

### Sampling strategy

A panel of 1213 *E. coli* isolates from four countries (Germany, UK, Spain, and Vietnam), obtained from five host species (human, pig, cattle, chicken, and wild boar) during three time periods (2003–2007, 2008–2012, and 2013–2018) from both healthy and diseased hosts were selected randomly from existing strain collections and newly collected isolates. Out of 120 possible strata (defined as a unique combination of country, host, time-period, and host health status), isolates were available for 42 strata. We included all isolates available per stratum if there were fewer than 30 isolates and performed a random selection of up to 30 isolates if more were available for a particular stratum. Potentially duplicate isolates that were part of an outbreak, isolated at a single location within a short timeframe, or from a single farm or a single individual were excluded. Only one isolate per individual was included in the analyses. Isolates included per stratum are shown in Additional file 2: Table S1.

*E. coli* isolates from Vietnam (*n* = 179) were retrieved from multiple collaborative studies at the Oxford University Clinical Research Unit, including the antimicrobial resistance surveillance in healthy humans (*n* = 62), chickens (*n* = 57), and pigs (*n* = 29) in Tien Giang province. *E. coli* from animal hosts were isolated from faecal material (faecal/rectal swabs or boot-swabs/hand-held swabs), whereas *E. coli* from diseased humans (*n* = 31) was isolated from blood and urine samples submitted to the Microbiology Laboratory at the Hospital for Tropical Diseases in Ho Chi Minh, Vietnam for routine diagnostic purposes.

Isolates from Spain (*n* = 233) were cultured from caecal samples collected in the frame of different research and surveillance projects. Samples from livestock were collected from the intestine of healthy animals at the abattoir immediately after slaughter with each sample, formed by specimens from one or more animals, representing an independent epidemiological unit (farm/flock). Samples from wild boar were collected from hunter-harvested wild boars during regular hunting seasons. All samples were refrigerated and sent to the VISAVET Health Surveillance Centre for the isolation and identification of *E. coli*.

Isolates from the UK (*n* = 174) were received from multiple sources including human hospitals (*n* = 64), university collections (*n* = 15), and private veterinary practices (*n* = 95). Human isolates (*n* = 64) were cultured from urine samples (*n* = 32) and faecal samples (*n* =32). Chicken isolates (*n* = 110) were obtained from diseased birds at post-mortem examination (*n* = 80) and faeces/cloacal swabs from healthy chickens (*n* = 30). Isolates were cultured as for routine diagnostic purposes.

Isolates from Germany collected at the Friedrich-Loeffler-Institut (FLI) (*n* = 30) were cultured from samples obtained in the context of different research and surveillance projects. Faecal samples from cattle were collected either from healthy animals present at the FLI facilities in Braunschweig or Jena or from the intestines of healthy animals at the abattoir with each sample taken from one animal from an independent farm. Human isolates (*n* = 170) were obtained from diseased patients with suspicion of infection with pathogenic *E. coli* (mainly STEC), sent to National Reference Centre at the Robert Koch Institute (RKI) from laboratories of primary diagnostics for confirmation and subtyping. The strains were chosen randomly within the predicted timeslot and the two categories of diseased with or healthy persons without symptoms. Other animals (pig, cattle and chickens) isolates (*n* = 202) were obtained from diagnostic labs, isolated from samples sent by vets because the corresponding animals were ill, whereas, for the healthy animals (pig, cattle and chickens) (*n* = 210), faecal samples were collected for routine diagnostic purposes.

### DNA extraction and sequencing

The DNA of the *E. coli* isolates from Germany was extracted using the QIAamp DNA Mini Kit (Qiagen) following the manufacturer’s instructions. The DNA concentration was evaluated fluorometrically by using Qubit^TM^ 2.0 fluorometer (Invitrogen, USA) and the associated Qubit^TM^ dsDNA HS Assay Kit (0.2–100 ng) and Qubit^TM^ BR Assay Kit (2–1000 ng), respectively. The libraries were generated using Nextera DNA library preparation (Illumina, https://www.illumina.com). The sequencing was performed using the Illumina MiSeq and HiSeq systems, generating 2 × 250 bp and 2 × 150 bp reads, respectively.

The DNA of the *E. coli* isolates from the UK was purified using a Promega DNA Wizard® genomic purification kit and quantified using Nanodrop. Libraries were generated using Nextera XT technology (Illumina), and DNA sequencing of isolates was performed at the Animal and Plant Health Agency (APHA, Surrey, UK, https://www.gov.uk/government/-organisations/animal-and-plant-healthagency) using an Illumina MiSeq system generating 2 × 150 bp reads.

For *E. coli* isolates from Spain, DNA was extracted using the DNA blood and tissue Qiagen kit according to the manufacturer’s instructions. The total amount of DNA was quantified using a Qubit fluorometer and frozen at – 20 °C until further analysis. Libraries were prepared using Nextera XT DNA Library preparation (Illumina), and DNA samples were sequenced using a MiSeq platform (2 × 300 cycle V3 Kit).

The DNA of the *E. coli* isolates from Vietnam was extracted using the Wizard Genomic DNA purification kit (Promega, Madison, WI, USA) following the manufacturer’s instructions. The concentration of the DNA was measured fluorometrically by using picogreen (Invitrogen). The sequencing was performed using an Illumina HiSeq 4000 system, which generates 2 × 150 bp reads.

### Quality control

Adapter sequences were removed from raw reads using flexbar v3.0.3 [58, 59] with trimming mode (-ae) ANY. Low-quality bases within raw reads (Phred score value < 20) were trimmed using a sliding window approach (-q WIN). FastQC v0.11.7 [60] and MultiQC v1.6 [61] were used for quality control before and after processing steps.

### Genome assembly and annotation

Adapter-trimmed reads were assembled using SPAdes v3.13.1 [62] using read correction. Scaffolds smaller than 500bp were discarded. QUAST v5.0.0 [63] was used to assess assembly quality using default parameters. Draft assemblies were excluded if the N50 was below an arbitrary value of 30 kbp or consisted of more than 900 contigs. Draft genomes were annotated using prokka v1.13 [64] with a genus-specific blast for *Escherichia.* Phylogroups were predicted using ClermonTyper v1.4.1 [65], and sequence types (STs) of the isolates were identified in silico using the Achtman seven gene MLST scheme using mlst (https://github.com/tseemann/mlst).

### Pan-genome and phylogenetic analysis

Roary v3.12.0 [66] was used to define the pan-genome of the population, using paralog splitting. The core genes were aligned using prank [67] on default parameters. The core gene alignment was used to construct the phylogenetic tree using RaxML 8.2.4 [68] with 100 bootstraps under a General Time Reversible (GTR) substitution model with the Gamma model of rate heterogeneity and Lewis ascertainment bias correction [69]. The core gene phylogeny was corrected for recombination using ClonalFrameML [70] using default parameters. BAPS clusters within the dataset were defined using hierBAPS [71, 72] based on the core gene alignment. The accessory gene clustering was performed using package Rtsne v0.15 [73, 74] with 5000 iterations and perplexity 15 in R v3.6.1. iTOL [75] and Microreact [76] were used to visualize the population structure in the context of available metadata. The function chisq.test from the MASS library [77] (v7.3-51.1) was used in R [78] (v3.5.2) to perform *χ*^2^-tests of independence between phylogenetic clusters and host species. Tests were carried out on the full dataset (14 phylogenetic clusters vs. five hosts and nine phylogroups vs. five host species).

### Genome-wide association study (GWAS)

We excluded the wild boar *E. coli* isolates from the GWAS analysis, because of their low number (*n* = 29). The GWAS was performed on *k-mers* as well as on the pan-genome matrix (i.e. gene presence and absence) generated from Roary.

First, assemblies were shredded into *k-mers* of 9–100 bases using FSM-lite (https://github.com/nvalimak/fsm-lite). The association of *k-mers* and gene presence/absence with host phenotype (i.e. pig, human, chicken, and cattle) was carried out using FaST-LMM, a linear mixed model implemented in pyseer whilst correcting for population structure and sampling bias [79]. To correct for population structure, a pairwise similarity matrix (based on patristic distances) generated from core-genome phylogeny (recombination free) was used with a FaST-LMM mixed model [79]. To account for sampling bias, a covariate matrix consisting of geographical location, year of isolation, and host health status was used.

GWAS analysis was carried out by comparing human hosts vs other hosts (i.e. pig, cattle, and chicken), cattle vs other hosts (i.e. human, chicken, and pig), chicken vs other hosts (i.e. human, pig, and cattle), and pig vs other hosts (i.e. cattle, chicken, and human) within the collection. To further reduce the false-positive associations and account for unbalanced groups, we employed a bootstrapping approach as implemented by Epping et al. (2021) [80]. Further details can be found in the additional file 1. *K-mers* and genes (presence-absence), which were significantly associated with the hosts in 90% of the runs, were retained. These *k-mers* were mapped to reference genomes (Additional file 2: Table S5) using a fastmap algorithm in bwa [79, 81]⁠. The genes significantly associated with a certain host in both GWAS approaches were considered further for downstream analysis.

### Prevalence of a human-associated *nan* gene cluster

All available *E. coli* genome assemblies in NCBI RefSeq were downloaded on November 29, 2019, using NCBI-genome-download (https://github.com/kblin/ncbi-genome-download). Using a custom ABRicate (https://github.com/tseemann/abricate) database, consisting of the nine genes of the novel human-associated *nan* gene cluster, all downloaded genomes (*n* = 17994) were scanned. STs for all the genomes were assigned as described above.

### Construction of mutants and phenotypic experiments

Mutants Δ*nan-9* (Amp^R^) and Δ*nanRATEK* of extra-intestinal pathogenic *E. coli* (ExPEC) strain IMT12185 (ST131; RKI 20-00501; Amp^R^) were constructed using the Datsenko-Wanner method [82]. The genomic DNA of the wild-type and the mutant strains was isolated using a QIAamp DNA Mini Kit (QIAGEN). Short read sequencing libraries were prepared using the Nextera XT DNA Library preparation kit (Illumina) and paired-end sequencing was performed on an Illumina MiSeq machine. The absence of the desired genes was confirmed by mapping sequencing reads on the most closely related complete reference genome (GCF_900520325.1) using Snippy 4.6.0.

Carbon utilization and chemical sensitivity of the deletion mutants and their parental strain were tested using a Biolog Phenotypic Array system, using the PM1 MicroPlate and the Gen III MicroPlate according to the manufacturer’s instructions.

### Growth curve analysis

*E. coli* strains were grown at 37 °C aerobically in lysogeny broth (LB) (10 g/l tryptone, 5 g/l yeast extract, 5 g/l NaCl, pH 7.5) or in minimal medium (MM). MM is M9 mineral medium (33.7 mM Na_2_HPO_4_, 22.0 mM KH_2_PO_4_, 8.55 mM NaCl, 9.35 mM NH_4_Cl) supplemented with 2 mM MgSO_4_ and 0.1 mM CaCl_2_. As carbon and energy source, either 27.8 mM [0.5% w/v] glucose, 6.47 mM [0.2% w/v] 5-N-acetylneuraminic acid (Neu5Ac) or 6.15 mM [0.1% w/v] N-glycolylneuraminic acid (Neu5Gc) (all purchased from Sigma-Aldrich, Taufkirchen, Germany) was added. If appropriate, the following antibiotics were used: ampicillin sodium salt (150 μg/ml) or kanamycin (50 μg/ml). For solid media, 1.5% agar (w/v) was added. For all growth experiments, bacterial strains were grown in LB medium overnight at 37 °C aerobically, washed twice in PBS and then adjusted to an optical density at 600 nm (OD_600_) of 0.005 in the desired liquid growth medium, or streaked on agar plates. Growth curves were obtained from bacterial cultures incubated at 37 °C with gentle agitation in 96-well microtitre plates containing 200 μl medium. The OD_600_ was measured by an automatic reader (Epoch2T; BioTek, Bad Friedrichshall, Germany) at appropriate time intervals as indicated.

## Supplementary Information

**Additional file 1:** **Fig. S1.** Distribution of 1,198 isolates and enrichment analysis: A) The plot represents the proportion of *E. coli* isolates isolated from hosts in four countries. The number above each plot indicates the total number of isolates per host. B) Phylogroups enriched with different hosts (Pearson residual > 0 represents positive correlation indicating the enrichment of certain host-species in distinct clusters at *p*-value < 2.2e^−16^). **Fig. S2.** Core genome phylogeny of *E. coli* isolates from our collection (*n*=1,198) and reference strains (*n*=146) from the ECOR collection, RefSeq and cryptic clades annotated with their phylogroups (phylogroups were determined by ClermonTyper v. 1.3). **Fig S3.** Core-genome phylogeny and Accessory-genome clustering of 1,198 *E. coli* isolates showing different BAPS clusters. Each BAPS on accessory genome clustering consist of the same strains as on the core-genome phylogeny. **Fig. S4.** Minimum-spanning tree of MLST profiles of 1,198 *E. coli* isolates. Left: the number of isolates constituting an ST; Right: the proportion of hosts in each ST. **Fig. S5.** Genetic surroundings of the human-associated *nan* gene cluster in the genomes of all isolates in which it was identified.

**Additional file 2:** **Table S1.** Metadata of 1,198 *E. coli* isolates. **Table S2.** Genes and genetic variants associated with different hosts that came as significant by both *k-mer* based as well as pan-genome based GWAS. **Table S3.** Prevalence of *ompP*, *arlC* and *ompT* in different STs in the RefSeq collection (*n*=17,994) and in our collection of *E. coli* isolates (*n*=1198). **Table S4.** Prevalence of the *iroBCDEN* gene cluster in sequence types and associated hosts. Details are shown for sequence types (STs) with at least ten isolates harboring *iroBCDEN*. **Table S5.** Genomes from our dataset used for annotating host-associated *k-mers*.

## Data Availability

The raw reads of the 1090 *E. coli* isolates sequenced in this study were submitted to NCBI SRA with the Bioproject accession number PRJNA739205 [83], and the SRA accession of 108 isolates [84], that was taken from other studies, was provided in Additional file 2: Table S1.

## Abbreviations

AMP: Antimicrobial peptide
AMR: Antimicrobial resistance
APEC: Avian pathogenic *Escherichia coli*
BAPS: Bayesian analysis of population structure
EDEC: Edema-disease *Escherichia coli*
ETEC: Enterotoxigenic *Escherichia coli*
ExPEC: Extraintestinal pathogenic *Escherichia coli*
GWAS: Genome-wide association study
LB: Lysogeny broth
ML: Maximum-likelihood
MM: Minimal medium
NCBI: National Center for Biotechnology Information
Neu5Ac: 5-*N*-acetylneuraminic acid
Neu5GC: 5-*N*-glycolylneuraminic acid
Sia: Sialic acids
ST: Sequence type
STEC: Shiga toxin-encoding *Escherichia coli*
UPEC: Uropathogenic *Escherichia coli*
