# Supplementary material for: Genome-wide association reveals host-specific genomic traits in Escherichia coli
Source: BMC Biol. 2023 Apr 11;21:76. doi: 10.1186/s12915-023-01562-w (PMC10088187; doi:10.1186/s12915-023-01562-w)

**GWAS method:**

For each “test-group” (comprise of Human or Chicken or Cattle or Pig isolates only) was compared with an equal number of isolates from “control-group”. The control-group were created by subsampling from “other-hosts” (excluding isolates in test-group).

For example:

To identify *k-mers* associated with Human host, the test-group (consisting of all human *E. coli* isolates, total no. of isolates = 327) were compared with other-hosts (group consist of chicken, cattle and pig *E. coli* isolates, total no. of isolates = 861). Before running the pyseer, a new control group is formed by subsampling the “other-hosts”. Hence, for run1 of pyseer, the phenotypic file was consisting of n=654 isolates (i.e., test-group = 327 + control-group = 327).

During the subsampling, the isolates in “other-hosts” were grouped based on their BAPS cluster. Then, a proportion of strains were randomly selected from each BAPS cluster. The same process was repeated for 100 runs of pyseer for human host. Which means for each run of pyseer the human *E. coli* isolates were compared with a new set of isolates in “control-group”.

Likewise, this was done for each host. The same method was used while running pyseer with pan-genome matrix. For each run a statistical significance threshold (i.e., *p-value* using Bonferroni correction) was estimated based on the number of unique patterns [84] and compared with lineage corrected pvalue (lrt-pvalue) for each *k-mer/gene* obtained from pyseer. In each run the k-mers/genes with effect-size (ß) > 0, lrt-pvalue < *p-value* were consider significantly associated with the host. Those k-mers/genes which came significant in 90% of the runs per host were retained.

The *p-value* threshold used to filter k-mers/genes.

| **Hosts** | ***p-vlaue* threshold *** | |
| --- | --- | --- |
|  | **GWAS: *Kmers*** | **GWAS: Pan-genome** |
| Human | 1.83x10^-09^ | 2.72x10^-06^ |
| Cattle | 2.08x10^-09^ | 2.83x10^-06^ |
| Chicken | 1.88x10^-09^ | 2.75x10^-06^ |

* the average *p-value* of 100 runs per host were shown in table.

***Supplementary Figures:***

**Fig. S1:** Distribution of 1,198 isolates and enrichment analysis: **A)** The plot represents the proportion of *E. coli* isolates isolated from hosts in four countries. The number above each plot indicates the total number of isolates per host. **B)** Phylogroups enriched with different hosts (Pearson residual > 0 represents positive correlation indicating the enrichment of certain host-species in distinct clusters at p-value <2.2e^-16^).


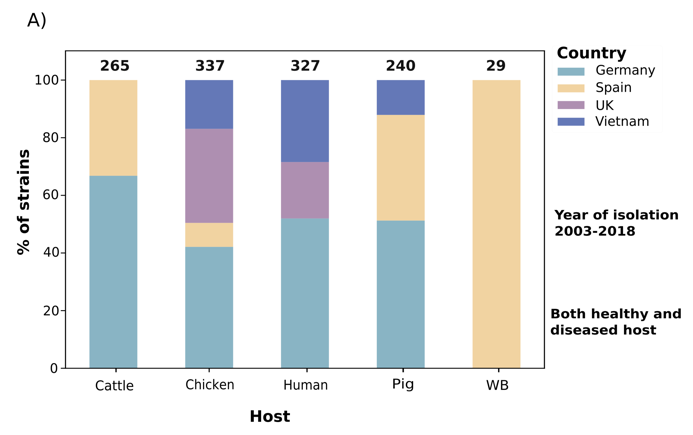

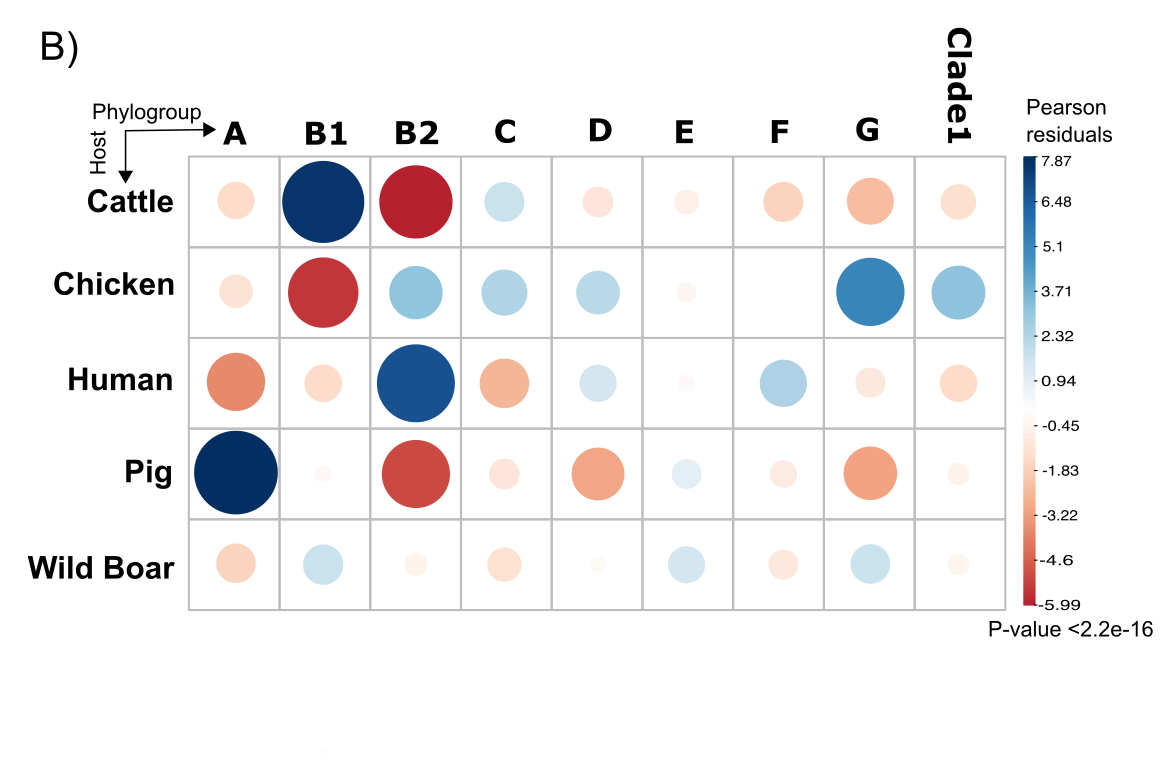


**Fig. S2:** Core genome phylogeny of *E. coli* isolates from our collection (n=1,198) and reference strains (n=146) from the ECOR collection, RefSeq and cryptic clades annotated with their phylogroups (phylogroups were determined by ClermonTyper v. 1.3).


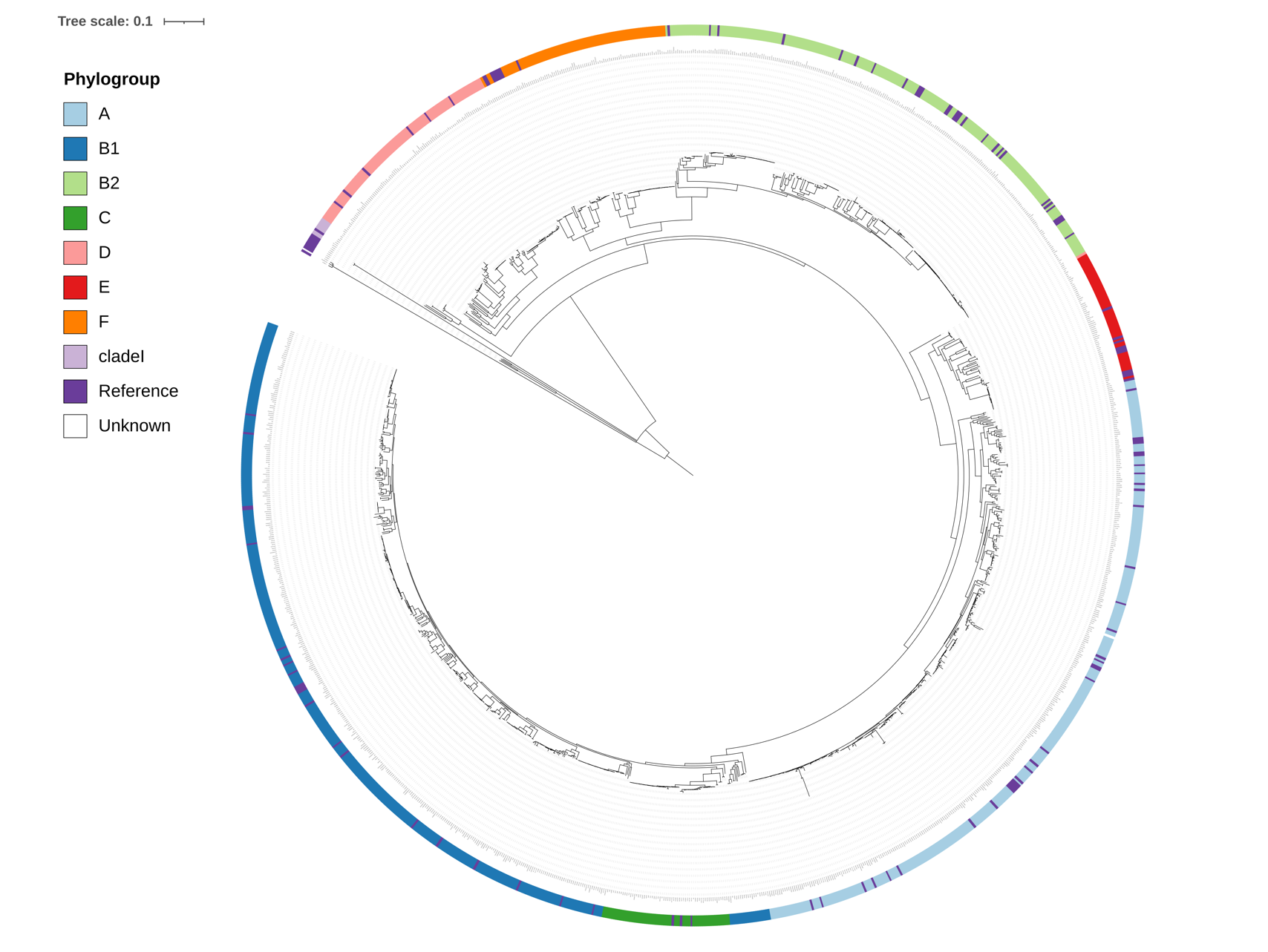


**Fig S3:** Core-genome phylogeny and Accessory-genome clustering of 1,198 *E. coli* isolates showing different BAPS clusters. Each BAPS on accessory genome clustering consist of the same strains as on the core-genome phylogeny.

**
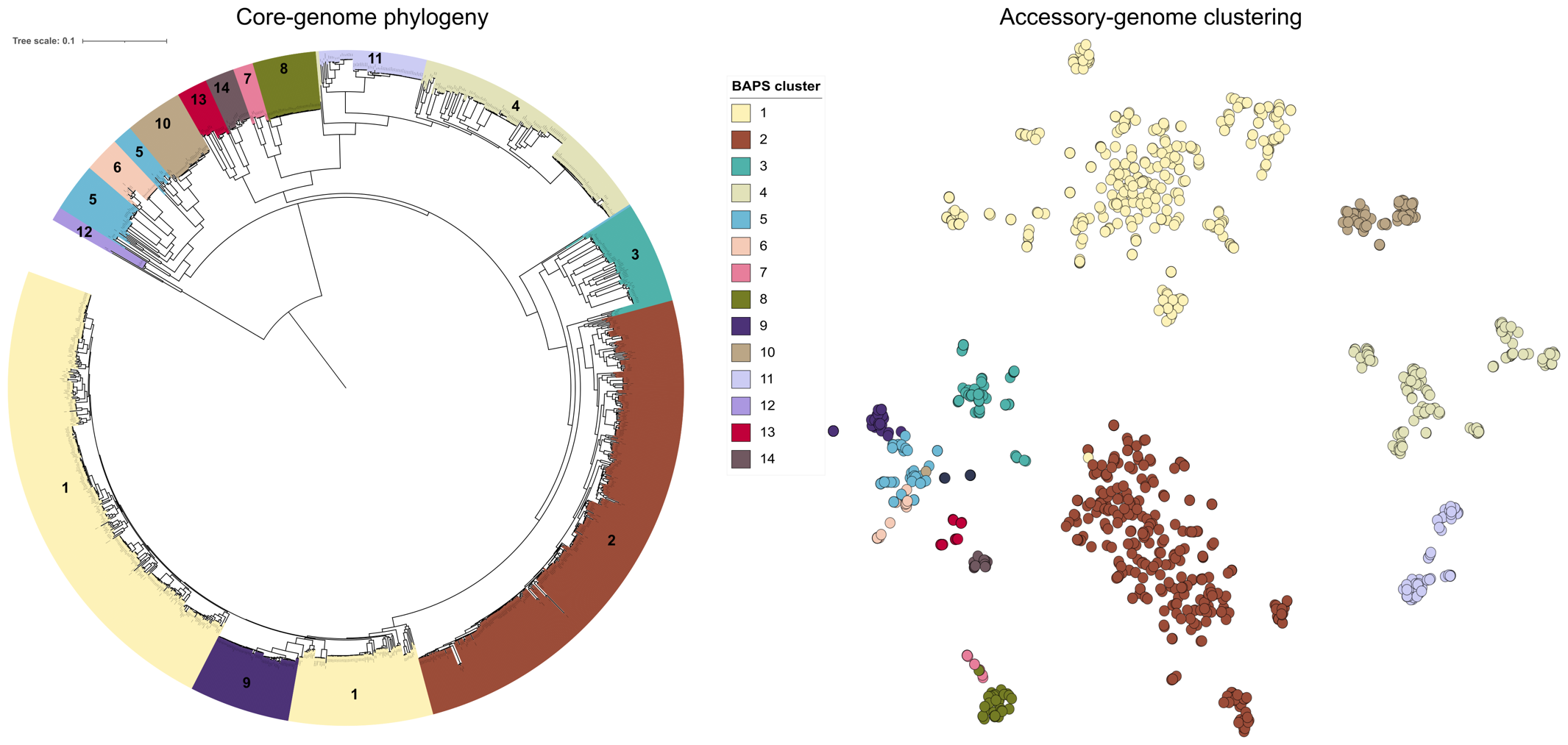
**

**Fig. S4:** Minimum-spanning tree of MLST profiles of 1,198 *E. coli* isolates. Left: the number of isolates constituting an ST; Right: the proportion of hosts in each ST.


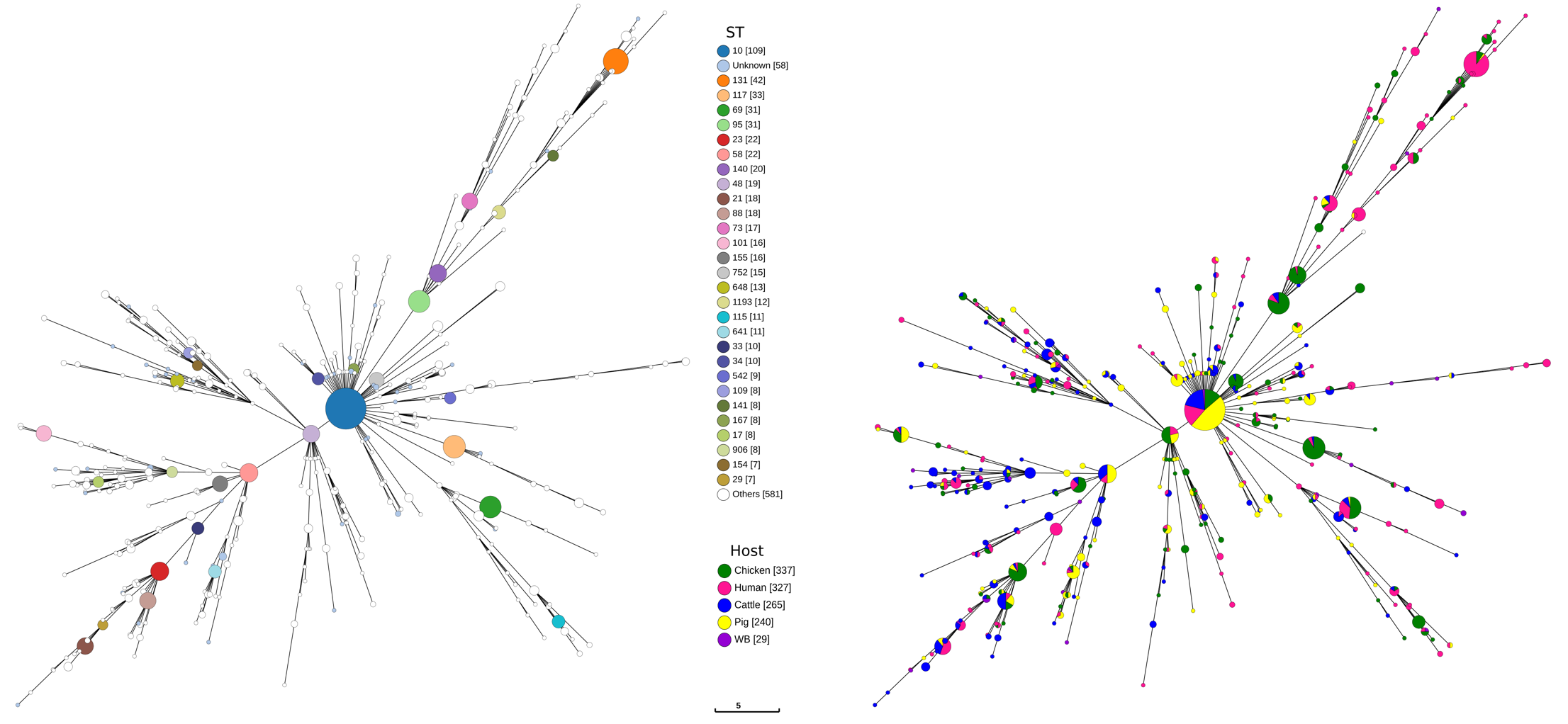


**Fig. S5:** Genetic surroundings of the human-associated *nan* gene cluster in the genomes of all isolates in which it was identified.


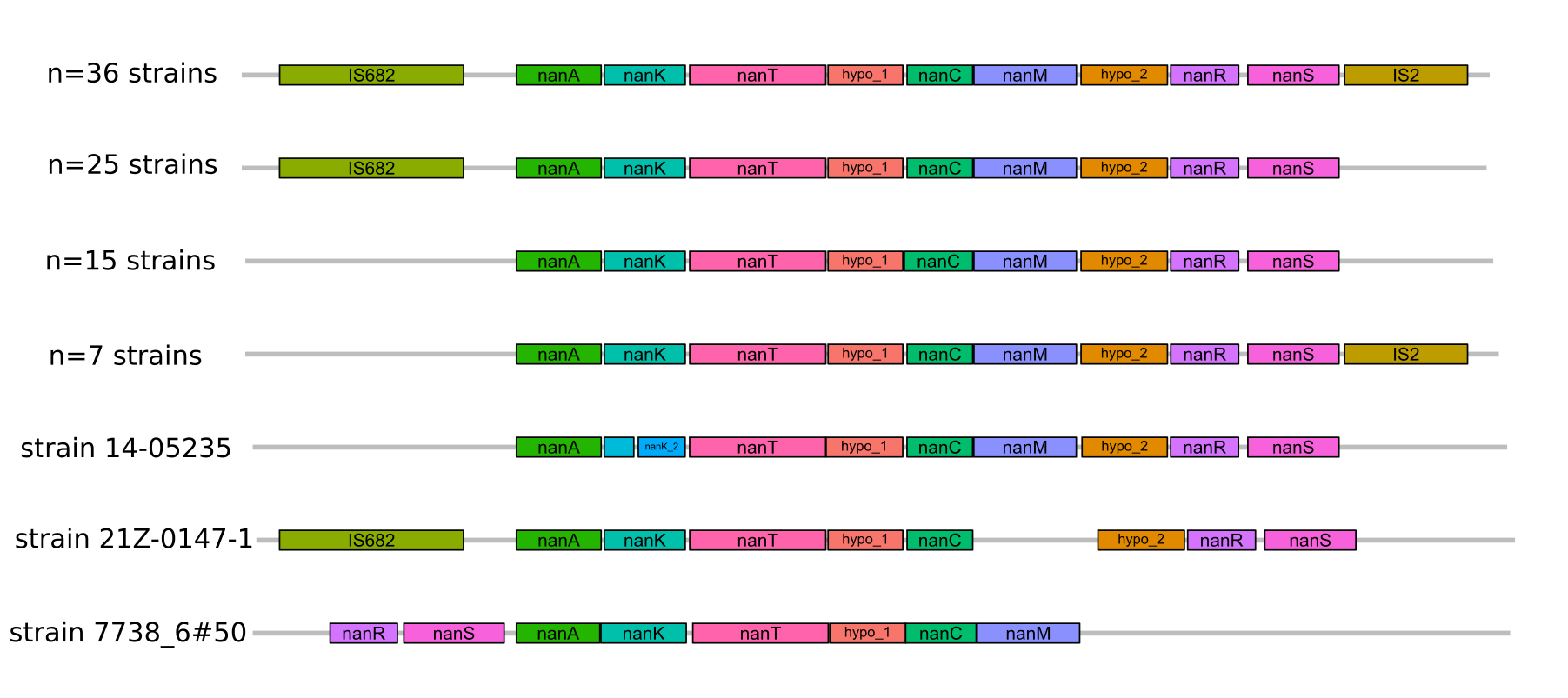

Supplement: Supplementary file 1 — Additional file 1: Fig. S1. Distribution of 1,198 isolates and enrichment analysis: A) The plot represents the proportion of E. coli isolates isolated from hosts in four countries. The number above each plot indicates the total number of isolates per host. B) Phylogroups enriched with different hosts (Pearson residual > 0 represents positive correlation indicating the enrichment of certain host-species in distinct clusters at p-value < 2.2e−16). Fig. S2. Core genome phylogeny of E. coli isolates from our collection (n=1,198) and reference strains (n=146) from the ECOR collection, RefSeq and cryptic clades annotated with their phylogroups (phylogroups were determined by ClermonTyper v. 1.3). Fig S3. Core-genome phylogeny and Accessory-genome clustering of 1,198 E. coli isolates showing different BAPS clusters. Each BAPS on accessory genome clustering consist of the same strains as on the core-genome phylogeny. Fig. S4. Minimum-spanning tree of MLST profiles of 1,198 E. coli isolates. Left: the number of isolates constituting an ST; Right: the proportion of hosts in each ST. Fig. S5. Genetic surroundings of the human-associated nan gene cluster in the genomes of all isolates in which it was identified. [file 12915_2023_1562_MOESM1_ESM.docx]
